# Understanding the Mechanism of Insulin and Insulin-Like Growth Factor (IGF) Receptor Activation by IGF-II

**DOI:** 10.1371/journal.pone.0027488

**Published:** 2011-11-28

**Authors:** Clair L. Alvino, Shee Chee Ong, Kerrie A. McNeil, Carlie Delaine, Grant W. Booker, John C. Wallace, Briony E. Forbes

**Affiliations:** School of Molecular and Biomedical Science, The University of Adelaide, Adelaide, Australia; University of North Carolina at Chapel Hill, United States of America

## Abstract

**Background:**

Insulin-like growth factor-II (IGF-II) promotes cell proliferation and survival and plays an important role in normal fetal development and placental function. IGF-II binds both the insulin-like growth factor receptor (IGF-1R) and insulin receptor isoform A (IR-A) with high affinity. Interestingly both IGF-II and the IR-A are often upregulated in cancer and IGF-II acts via both receptors to promote cancer proliferation. There is relatively little known about the mechanism of ligand induced activation of the insulin (IR) and IGF-1R. The recently solved IR structure reveals a folded over dimer with two potential ligand binding pockets arising from residues on each receptor half. Site-directed mutagenesis has mapped receptor residues important for ligand binding to two separate sites within the ligand binding pocket and we have recently shown that the IGFs have two separate binding surfaces which interact with the receptor sites 1 and 2.

**Methodology/Principal Findings:**

In this study we describe a series of partial IGF-1R and IR agonists generated by mutating Glu12 of IGF-II. By comparing receptor binding affinities, abilities to induce negative cooperativity and potencies in receptor activation, we provide evidence that residue Glu12 bridges the two receptor halves leading to receptor activation.

**Conclusions/Significance:**

This study provides novel insight into the mechanism of receptor binding and activation by IGF-II, which may be important for the future development of inhibitors of its action for the treatment of cancer.

## Introduction

**Figure 1:**
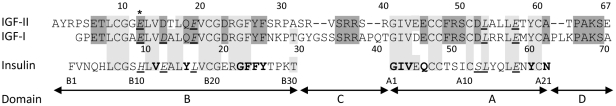
The amino acid sequence alignment of IGF-II, IGF-I, and insulin. Insulin residues important for IR binding and defined as site 1 residues (ValB12, TyrB16, GlyB23, PheB24, Phe B25, TyrB26, GlyA1, IleA2, ValA3, GlnA5, TyrA19, AsnA21) are shown in bold type and those defined as site 2 residues (HisB10, GluB13, LeuB17, SerA12, LA13, Glu17) are underlined and in italics [18]. IGF-I and IGF-II site 2 residues are underlined and in italics. Conserved residues are boxed in light gray, residues conserved between IGF-II and IGF-I are boxed in dark gray and the domain structure is below. Residue Glu 12 of IGF-II mutated in this study is highlighted with an *asterisk*.

The insulin-like growth factors (IGF-I and IGF-II) share sequence and structural similarities with insulin. IGFs have four domains in the order B, C, A, and D from the N terminus (Figure 1) and three alpha helices making up the core structure. Insulin is produced as a propeptide which, when processed to the mature form, only has the B and A domains on separate chains linked as a dimer by disulphide bonds and having a similar helical structure to the IGFs. In contrast to the critical role of insulin in metabolic control the IGFs act via the type 1 IGF receptor (IGF-1R) to promote cell proliferation, survival and differentiation. IGFs are essential for normal growth and development, and perturbation of IGF-I expression is associated with acromegaly (increased) [1] or short stature (decreased) [2]. Disruption of IGF-II imprinting during development is associated with overgrowth in Beckwith-Wiedemann syndrome, whereas reduced paternal allele expression results in growth retardation in Silver-Russell syndrome [3]. Furthermore, IGFs acting via the IGF-1R play a major role in promoting cancer cell growth and survival [4]. Therefore understanding the mechanism of receptor activation will aid in the understanding of the role of these ligands in disease.

The insulin receptor (IR) exists in two isoforms which arise by alternative splicing of exon 11 [5], [6]. The IR-B isoform binds insulin with high affinity. The IR-A isoform, which lacks the 12 amino acids normally encoded by exon 11, not only binds insulin with high affinity but can also bind IGF-II, albeit with a 6 fold lower affinity [7], [8]. Whereas insulin promotes metabolic signalling via both isoforms, interestingly, IGF-II predominantly promotes activation of mitogenic signalling outcomes such as protection from apoptosis via the IR-A [9], [10]. Expression of IGF-II and IR-A in foetal tissues is supportive evidence for the role of these molecules in development [6]. Furthermore, expression of both is upregulated in many cancer cells and tissues suggesting a role in promoting cancer cell growth and survival [5], [6] and explaining the resistance of certain cancer cells to inhibition of growth by anti-IGF-1R antibodies [11].

Activation of the IR and IGF-1R leads to signalling via two main pathways. Following activation of the tyrosine kinase domain the receptors undergo autophosphorylation, which promotes binding of adapter molecules such as the insulin-receptor substrates (IRS-1 and -2) and Shc. These proteins then led to activation of the phosphatidylinositol 3-kinase (PI3K/Akt) and the extracellular signal-regulated kinase (ERK/MAPK) cascades [12].

The IR and IGF-1R are structurally similar, each made of homodimers with a 2α2β subunit configuration. The ectodomain adopts a folded over conformation with two potential ligand binding pockets [13]. Within a binding pocket there are two distinct binding surfaces: the L1 and FnIII-2 insert domains from opposite receptor halves form one binding surface (site 1) [13], [14], [15], [16] and residues within the FnIII-1 and FnIII-2 domains form the other surface (site 2) [17]. Each ligand binds the receptor with a stoichiometry of 1∶1 at physiological concentrations. Binding of a second ligand molecule in the other binding pocket accelerates the dissociation of the first resulting in a negative cooperativity binding mechanism [18], [19].

As there is currently no structure of these ligand:receptor complexes our understanding of the mechanism of binding is derived from mutagenesis and cross-linking studies. There is a similar overall binding mechanism for the insulin:IR and the IGF:IGF-1R interactions [20], although there is an additional contact made between the IGF-I C-domain and the IGF-1R cysteine rich (CR) domain [15], [21]. Insulin has two distinct surfaces (sites 1 and 2), which contact the IR. The site 1 insulin binding surface includes residues ValB12, TyrB16, TyrB26, and ValA3 (residues corresponding to the dimerisation interface) and these contact residues within the IR site 1 described above. Insulin binding site 2 includes residues HisB10, GluB13, LeuB17, SerA12, LeuA13 and GluA17 (Figure 1) [19]. Two similar sites also exist on IGF-I and IGF-II although there are subtle differences in the relative contribution of each residue to the receptor interactions [22], [23] (Figure 1).

So far there is little direct evidence of the mechanism by which ligand binding results in receptor activation. Mutagenesis studies suggest that the insulin site 2 residue HisB10 plays an important role in IR activation and in particular mitogenic signalling. In this study we aim to investigate our proposal that the equivalent site 2 residue of IGF-II (Glu12) may also be important for receptor binding and activation. We describe a series of Glu12 mutants which act as partial IGF-1R and IR agonists. We provide evidence that site 2 residue Glu12 bridges the two receptor halves leading to receptor activation by comparing receptor binding affinities, abilities to induce negative cooperativity and potencies in receptor activation. These findings provide strong evidence to support the current model of receptor activation, which incorporates a bridging event to initiate receptor activation and downstream signalling. This study has therefore lead to a greater understanding of the mechanisms of IGF-II binding and activation of the IGF-1R and IR-A.

## Results

### Production and Structural Characterization of IGF-II Mutants

Six IGF-II mutants with single amino acid substitutions (to Ala, Asp, His, Lys, Gln and Arg) at the Glu12 position were produced to analyse the contribution of this residue to receptor binding and activation. All mutants were purified following successful expression in *E.coli* and were shown by mass spectrometry to be of the correct mass. Expression and processing of Glu12Arg IGF-II was considerably less efficient than for IGF-II and the other mutants. The far-UV CD spectra for all the mutants, Glu12Arg IGF-II included, were essentially identical to that of IGF-II (Figure S1), indicating that the substitutions had little overall effect on secondary structure.

### IGF-1R and IR-A Binding

#### Binding to Solubilised Receptors

**Figure 2:**
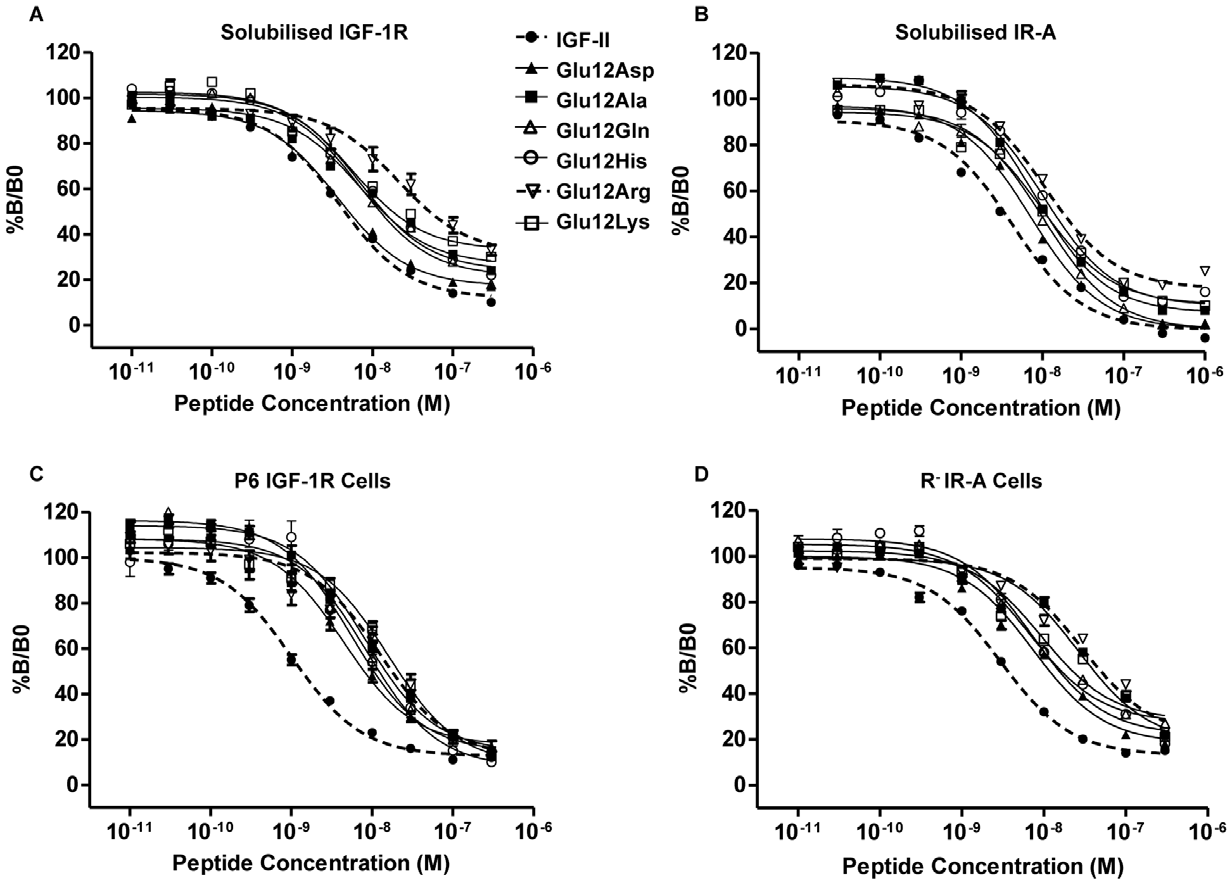
Competitive binding of IGF-II and Glu12 mutants to IGF-1R and IR-A either solubilised or on intact cells. Immunocaptured solubilised IGF-1R and IR-A (*A* and *B*) or P6 IGF-1R and R^−^IR-A cells (*C* and *D*) were incubated with Eu-IGF-II in the presence or absence of increasing concentrations of IGF-II (•), Glu12Asp IGF-II (▴), Glu12Ala IGF-II (▪), Glu12Gln IGF-II (▵), Glu12His IGF-II (○), Glu12Arg IGF-II (▿) or Glu12Lys IGF-II (□). Results are expressed as a percentage of binding in the absence of competing ligand (%B/Bo), and the data points are the mean±SEM of at least three separate experiments with each point performed in triplicate. Error bars are shown when greater than the size of the symbols.

The affinities of the Glu12 IGF-II mutants for detergent solubilised, immunocaptured IGF-1R and IR-A were measured in competition binding assays (Figure 2A and 2B). The IC_50_ values derived from these assays are presented for each of the mutants as values relative to IGF-II in Table 1. Alanine mutagenesis studies previously demonstrated that meaningful changes in affinity resulting from single amino acid substitutions range from 2- to 100-fold [24]. We therefore consider here only >2-fold changes in affinity to be significant (fold change refers to the ratio of the IC_50_ value of the analogue to the IC_50_ value of IGF-II). By this definition, five of the six mutants (all but Glu12Asp IGF-II) had significantly lower affinities for the solubilised IGF-1R than IGF-II (Figure 2A). Glu12Lys, Glu12Ala, Glu12His and Glu12Gln IGF-II all had similar binding affinities that were 36–43% of IGF-II, while replacement of Glu12 with Arg caused the greatest disruption to binding (5.2-fold worse than IGF-II). In contrast, only two of the six mutants displayed significant decreases in affinity for the solubilised IR-A (Figure 2B, Glu12Lys and Glu12Arg IGF-II with 2.3- and 2.4- fold lower affinities than IGF-II respectively).

**Table 1:** Relative binding of IGF-II and the IGF-II analogues for the IGF-1R and IR-A.

Affinity (%IGF-II ± SEM)					
	IGF-1R	IR-A	P6 IGF-1R	R^−^IR-A^b^	R^−^IR-A/P6 IGF-1R^c^
**IGF-II** a	100 (3.6±0.6 nM)	100 (4.2±1.2 nM)	100 (1.1±0.3 nM)	100 (3.1±1.7 nM)	1.0
**Glu12Ala**	40.8±8.9***	57±9**	14.2±3.6***	10.4±2.2***	0.7
**Glu12Asp**	73.3±12.4^ns^	58±6*	23.2±5.7***	39.7±14.9***	1.7
**Glu12His**	40.6±13.3***	52±16**	12±2.1***	34.2±9.6***	2.9
**Glu12Lys**	43.4±12.2***	44±5***	6.9±2***	32.1±7.4***	4.7
**Glu12Gln**	35.9±10.3***	56±14**	23±6.8***	23.6±9.7***	1.0
**Glu12Arg**	17.7±1.6***	42±18***	6.6±1.5***	8.6±4.5***	1.3

a Binding affinities of the analogues relative to IGF-II were derived from the IC50 values. Relative binding is expressed as a percent of IGF-II ± S.E. The IC50 of IGF-II for each assay is shown in parentheses.

b Binding to R^−^IR-A = binding to R^−^IR-A cells, IR-A = to immunocaptured IR-A, P6 IGF-1R = to BALB/c3T3 cells overexpressing the IGF-1R, IGF-1R = to immunocaptured IGF-1R.

c R^−^IR-A/P6 IGF-1R is the ratio of IC50 values for binding to the R^−^IR-A cells versus P6 IGF-1R. Data is derived from at least 3 separate experiments performed in triplicate.

*** = p<0.001,

** = p value 0.001 to 0.01,

* = p value 0.01 to 0.05, ns = not significant when compared to IGF-II.

#### Binding to IGF-1R and IR-A on the cell surface

We and others have previously shown that in some cases the effect of mutations of insulin [25] or IGF [22], [23] on their receptor binding (including Glu12Ala in IGF-II [22]) is greater when measured on receptors within the cell membrane than on solubilised receptors. In the present study therefore, we also measured the IGF-1R and IR-A binding affinities of the mutants (Figure 2C and 2D) using P6-IGF-1R (BALB/c3T3 cells overexpressing the human IGF-1R) [26] and R^−^IR-A cells (IGF-1R negative (R^−^) cells overexpressing the IR-A) [8]. As expected all six mutants exhibited much lower affinities for membrane bound IGF-1R (Figure 2C, 4.3–15.1 fold lower than IGF-II) and IR-A (Figure 2D, 2.5–11.6 fold lower than IGF-II) compared to the equivalent solubilised receptors (1.36–5.6 fold and 1.72–2.38 fold lower than IGF-II for the soluble IGF-1R and IR-A respectively). Interestingly, Ala substitution was quite detrimental when binding to both membrane-bound IGF-1R (Figure 2C) and IR-A (Figure 2D) (10.4% and 14.2% of IGF-II respectively), whereas it had relatively little effect on binding to immunocaptured solubilised receptors (Table 1). For P6-IGF-1R binding, conservative substitutions of Glu12 with Asp or Gln resulted in 4.3-fold decreases in affinities relative to IGF-II (Figure 2C). Substitutions with the increasingly basic amino acids His, Lys and Arg were the most detrimental to IGF-1R binding with an 8.3-, 14.5- and 15.2 fold decrease in affinity relative to IGF-II, respectively. The effect of these mutations on IR-A binding did not exactly parallel their effects on IGF-1R binding (Figure 2D). Substitutions with Asp, Gln, His and Lys all had a similar effect on R^−^IR-A binding (24–40% of IGF-II), with His and Lys being tolerated on this receptor better than on the IGF-1R in the membrane (R^−^IR-A/P6 IGF-1R ratio of 2.9 and 4.7 respectively, see Table 1). Arg and Ala substitutions caused the greatest negative effect on R^−^IR-A binding with 10- and 12-fold decreases in affinity compared to IGF-II, suggesting size and charge may play a role in optimal binding.

### Dose-response Curves for Negative Cooperativity

**Figure 3:**
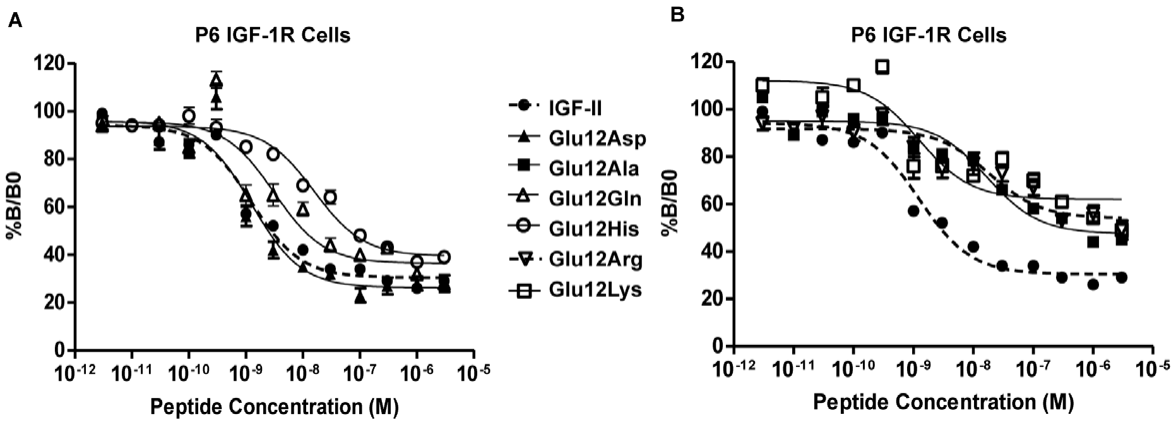
Dose-response curves for negative cooperativity. Accelerated dissociation of prebound Eu-IGF-II in the presence of increasing concentrations of (*A*) IGF-II (•), Glu12Asp IGF-II (▴),Glu12Gln IGF-II (▵), Glu12His IGF-II (○) and (*B*) IGF-II (•), Glu12Ala IGF-II (▪), Glu12Arg IGF-II (▿) or Glu12Lys IGF-II (□) from the IGF-IR on P6 IGF-1R cells. Results are expressed as a percentage of binding in the absence of competing ligand (%B/Bo) after 30 min, and the data points are the mean±SEM of three assays with each concentration measured in triplicate. Error bars are shown when greater than the size of the symbols. Curves are separated into two graphs for clarity.

While there are two potential binding pockets in each receptor, IGF-1R and IR binding studies indicate that only a single ligand molecule bridges a pair of binding surfaces within a single pocket at a time. The model of IGF-1R and IR binding proposed by De Meyts [19] suggests that a second ligand molecule, upon partial dissociation of the first (pre-) bound ligand, is able to bridge the alternate binding site and thereby accelerates the dissociation of the first ligand (defined as negative cooperativity). To assess the effect of Glu12 substitutions on negative cooperativity we measured the ability of each analogue to accelerate the dissociation of pre-bound europium-labelled IGF-II (Eu-IGF-II) in P6-IGF-1R cells. Dissociation of Eu-IGF-II tracer from the IGF-1R by unlabeled IGF-II produced a sigmoid dose response curve for negative cooperativity with 26% of tracer remaining bound after 30 minutes (Figure 3A and 3B, 50%B/B_o_ at 4 nM). Interestingly, the dose-response curve for Glu12Asp was indistinguishable from that of IGF-II (Figure 3A) despite the 4-fold decrease in affinity of Glu12Asp for P6-IGF-1R (Table 1). Glu12Gln had a ∼2.5 fold decrease in potency (indicated by the right shift in the dose-response curve in Figure 3A, 50% B/B_o_ reached at 10.5 nM) in line with its reduced affinity for the IGF-1R, and it did result in the same maximal acceleration of dissociation of Eu-IGF-II (28% of tracer remaining) as IGF-II. Glu12His (Figure 3A), Glu12Ala (Figure 3B) and Glu12Arg (Figure 3B) (in that order) showed further reduced potencies for negative cooperativity compared to IGF-II as evidenced by the rightward shift of these curves and a decrease in maximal dissociation of tracer (40–50% of tracer remaining bound). Glu12Lys was also very poor at accelerating dissociation of the Eu-IGF-II (Figure 3B). The reduced efficacy of IGF-II mutants in these assays is consistent with a reduced ability to bridge the alternate binding pocket. Dose-response curves for negative cooperativity were unable to be obtained for IGF-II and the IR-A on R^−^IR-A cells. The amount of tracer bound did not provide a suitable window to discern the differences between IGF-II and Glu12 IGF-II mutants. We suspect this is due to the lower level of receptor expression on these cells than on the P6-IGF-1R cells used for the IGF-1R assays and are exploring alternative experimental systems to allow these measurements with the IR-A in the future.

### IGF-1R and IR-A Kinase Receptor Activation Assays

**Figure 4:**
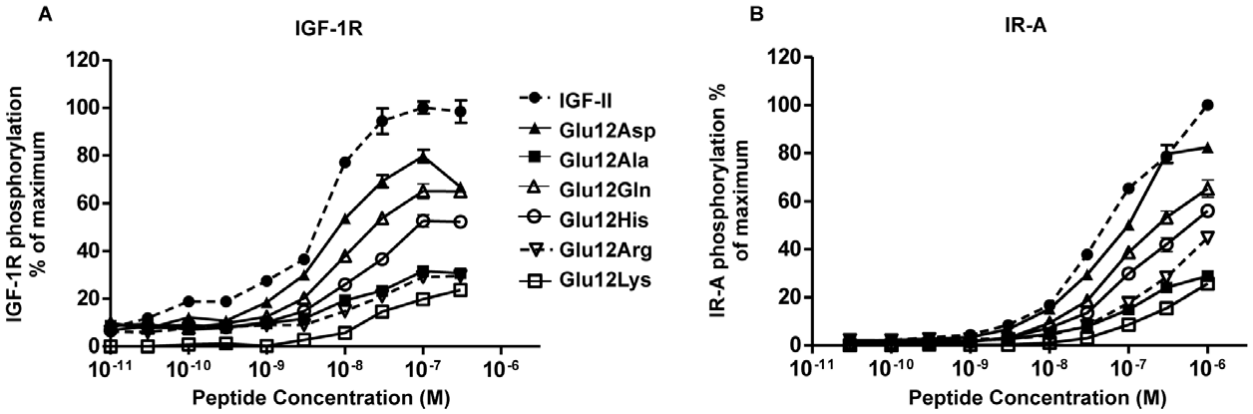
Activation of the IGF-1R and IR-A by IGF-II and Glu12 mutants. P6-IGF-1R cells (*A*) and R^−^IR-A cells (*B*) were serum starved for 4 h and then incubated with increasing concentrations of IGF-II (•), Glu12Asp IGF-II (▴), Glu12Ala IGF-II (▪), Glu12Gln IGF-II (▵), Glu12His IGF-II (○), Glu12Arg IGF-II (▿) or Glu12Lys IGF-II (□) for 10 min. Solubilised IGF-1R (*A*) and IR-A (*B*) were immunocaptured, and phosphorylated tyrosines were detected with Eu-PY20. Receptor phosphorylation is expressed as a percentage of the maximal phosphorylation induced by IGF-II. The data points are means±SEM of three assays with each concentration measured in triplicate. Error bars are shown when greater than the size of the symbols.

Activation of the IGF-1R on P6-IGF-1R cells and IR-A on R^−^IR-A cells by Glu12 mutants was assessed by measuring their capacities to stimulate total receptor tyrosine phosphorylation in kinase receptor activation assays. The dose-response curve for IGF-II-induced stimulation of IGF-1R (Figure 4A) in P6-IGF-1R cells was bell-shaped, with maximal phosphorylation at 100 nM IGF-II and self-antagonism apparent at higher concentrations. The bell-shaped activation curve is indicative of a bivalent bridging mechanism [27]. A similar curve has also been observed for IGF-I activation of the IGF-1R [8], [23] and is seen for insulin activating the IR [8], [27]. In the present study, a bell-shaped curve was not observed for IGF-II-induced phosphorylation of the IR-A (Figure 4B) within the concentration range explored and maximal phosphorylation was still being approached at 1 µM.

For both the IGF-1R (Figure 4A) and IR-A (Figure 4B) Glu12Asp was only slightly less potent than wild-type IGF-II (a rightward shift of ∼2 fold and >80% of maximal response) in its ability to activate receptor phosphorylation. The remaining mutants displayed reduced abilities to activate receptor phosphorylation in terms of both rightward shift of the curve and maximal response (decreased height of the curve). Glu12Gln induced phosphorylation of both receptors to 65% of the maximum seen for IGF-II and was slightly more potent than Glu12His. Glu12Ala and Glu12Lys were poor activators of IGF-1R and IR-A achieving just 24–32% of the maximum seen for IGF-II (Figure 4A and 4B respectively). The ability of Glu12Arg to activate both receptors was also significantly impaired and this was more so on the IGF-1R (maximal response 30% of IGF-II, Figure 4A) than the IR-A (maximal response 45% of IGF-II, Figure 4B).

Interestingly, the ability of each analogue to activate the receptors in terms of both rightward shift of the curve and maximal response essentially mirrored the relative potencies in the dose-response curves for negative cooperativity. However, we noticed a disparity for some mutants between receptor binding affinities and activity in both kinase receptor activation assays and dose response curves for negative cooperativity. For example, Glu12Ala IGF-II, with only a 7 fold lower IGF-1R binding affinity than IGF-II, required 100 fold more (300 nM) to achieve the same level of IGF-1R phosphorylation as IGF-II (ie a rightward shift when comparing concentrations required to achieve 30% phosphorylation and accompanied by only a 32% maximal response). The same could be said for Glu12Lys and Glu12Arg IGF-II. Also, all three showed a disparity in IR-A binding affinity and receptor activation activity. For example, Glu12Lys was a significantly weaker agonist of the IR-A in these assays than Glu12His (Figure 4B) despite their nearly identical IR-A binding affinities (Table 1). In addition, Glu12Ala bound IR-A with 10 fold lower affinity than IGF-II but 30 fold more Glu12Ala was required to achieve the same level of IGF-1R phosphorylation as IGF-II (ie a rightward shift when comparing concentrations required to achieve 30% phosphorylation accompanied by only a 29% maximal response). This is in contrast to IGF-I which, despite poor IR-A binding affinity (14 fold lower than IGF-II [8]), was able to activate the IR-A better than Glu12Ala IGF-II (Figure S2).

Finally, as Glu12Lys had less of an effect on IR-A binding affinity than IGF-1R binding affinity (32% versus 6.9% of IGF-II affinities respectively) it was a surprise to see an even greater detrimental effect on IR-A activation than expected (Figure 4B) Glu12Lys activated both the IR-A and IGF-1R to similar extents and only to 25% maximal response). In conclusion, Glu12Ala, Glu12Lys and Glu12Arg IGF-II exhibit a greater than expected decrease in ability to activate both IGF-1R and IR-A compared to their binding affinities for these receptors.

### Phosphorylation of the downstream signalling molecule Akt

**Figure 5:**
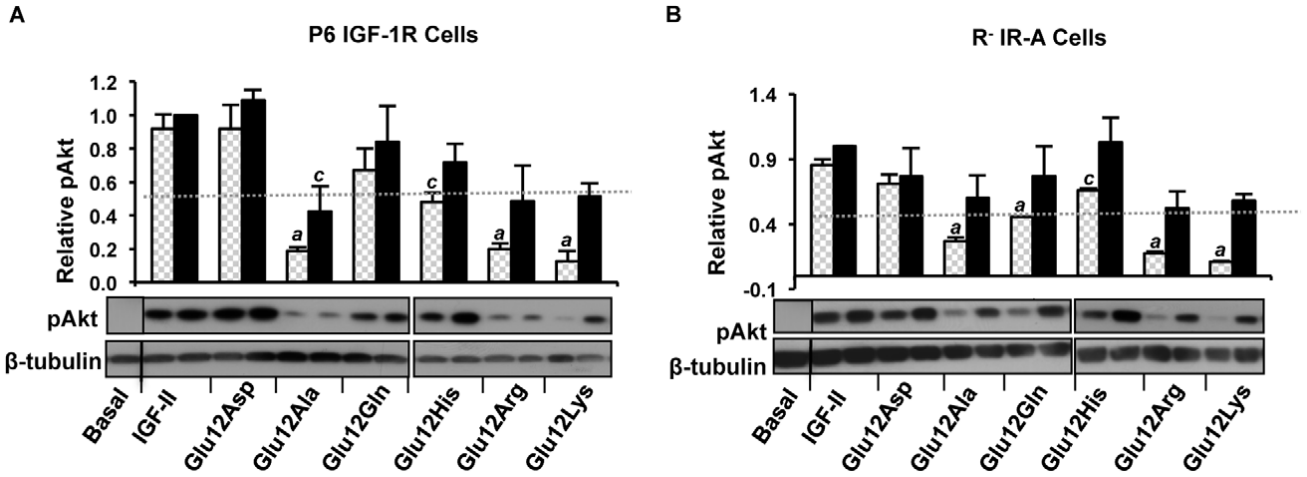
Induction of Akt phosphorylation upon IGF-1R and IR-A activation by IGF-II and Glu12 mutants. Serum-starved P6 IGF-1R (*A*) and R^−^IR-A cells (*B*) were treated with IGF-II, Glu12Asp IGF-II, Glu12Ala IGF-II, Glu12Gln IGF-II, Glu12His IGF-II, Glu12Arg IGF-II or Glu12Lys IGF-II at 10 nM (hatched bars) or 100 nM (solid bars) for 10 min. Whole-cell lysates were prepared and subjected to SDS-PAGE and then immunoblotted for phosphorylated Akt (pAkt). Representative blots are shown in the lower panels, and lanes 1–9 are 10–15 are from separate blots in both *A* and *B*. Each blot included lanes from cells untreated (basal) and treated with 100 nM IGF-II. In upper panels densitometry of three independent experiments ± SEM are shown as a column graph. Relative pAkt levels are expressed as a fraction of the level detected when cells were stimulated with 100 nM IGF-II. In each case pAkt was expressed as a fraction of the loading control (β-tubulin). *a* = p value<0.001 , *b* = p value 0.001 to 0.01, *c* = p value 0.01 to 0.05 when compared to IGF-II at the same concentration.

In order to examine signalling downstream of the receptors, activation of Akt in response to IGF-II and mutants was measured in P6-IGF-1R (Figure 5A) and R-IR-A cells (Figure 5B). Activated Akt (as measured by phosphorylation of Akt Ser473) was barely detectable and not quantifiable under basal conditions (i.e. serum free media) in either cell line. As shown in Figure 5A and 5B 10 nM IGF-II stimulated activation of Akt via both the IGF-1R and IR-A (as measured by phosphorylation of Ser473) to the same extent as 100 nM IGF-II. With the exception of Glu12Ala treatment of P6-IGF-1R cells (43% of IGF-II, p>0.01), there was no significant difference between the abilities of the Glu12 mutants and IGF-II to activate Akt when 100 nM was used to stimulate both P6-IGF-1R (Figure 5A) and R^−^IR-A cells (Figure 5B). However, when the P6-IGF-1R or R^−^IR-A cells were stimulated with 10 nM Glu12Arg, Glu12Ala or Glu12Lys IGF-II Akt activation was significantly reduced 3.3- to 7.2-fold compared with 10 nM IGF-II (Figure 5A and 5B respectively). Ten nanomolar Glu12Asp, Glu12Gln and Glu12His induced Akt activation to no less than half that seen with 10 nM IGF-II via both receptors. Thus, Akt activation correlates with ability to activate the receptors in the KIRA assays.

## Discussion

IGF-II binds to the IGF-1R and the IR-A and activates mitogenic signalling leading to cell proliferation and survival. Receptor binding involves a two site binding mechanism [22]. With a series of six novel IGF-II mutants we have demonstrated the importance of IGF-II residue Glu12 in IGF-1R and IR-A binding and activation and have shown that both the size and charge of the Glu sidechain at this position are important for achieving high affinity binding of IGF-II to both receptors.

In this study introduction of a positive charge at position 12 of IGF-II (as is found in the equivalent position in insulin (HisB10)) resulted in a lower affinity for both the IGF-1R and IR-A. A positive charge at the same position in IGF-I (Glu9Lys) also results in a lower affinity for the IGF-1R [28]. Conversely, substitution of the insulin residue HisB10 with a negatively charged amino acid (Asp or Glu) leads to an increase in IR and IGF-1R binding affinity and is associated with an enhanced mitogenic activity [29], [30], [31]. This suggests that a negative charge at this position in all three ligands is preferable when striving for the highest receptor binding affinity. However, the HisB10 found in insulin has apparently been selected for to achieve optimal metabolic signalling via the IR and confers to insulin the property of Zn^2+^ coordination in the hexameric form.

Interestingly the Glu to Lys or His charge reversals at position 12 of IGF-II had more effect on binding to the membrane bound IGF-1R (8- to 14-fold lower than IGF-II) than IR-A (3-fold lower than IGF-II). We conclude that while both receptors display a preference for negative charge at the Glu12 position, the IR-A displays a greater tolerance of positive charge here than the IGF-1R. This points to a difference in the nature of the second binding surface (i.e. that encountered by Glu12) on the IGF-1R and IR-A. Differences in IGF-1R and IR site 2 regions was previously demonstrated by Benyoucef *et al.* [32] in the context of an IGF-1R:IR-A hybrid receptor, which normally has low affinity for insulin. Introduction of the IR residues 325–524 (incorporating some of binding site 2) into the corresponding region of the IGF-1R half of the hybrid led to a 20-fold increase in insulin binding affinity. Recently specific IR residues contributing to site 2 binding were identified by site-directed mutagenesis [17] and some of these residues are not conserved in the IGF-1R sequence. Further mutagenesis studies would be required to identify which specific residues within the IGF-1R and IR-A site 2 regions are responsible for these differences in affinities for the Glu12Lys and Glu12His mutants.

It is clear that the effect of mutating Glu12 in IGF-II (or the equivalent residues in insulin [25] and IGF-I [23]) is greater on membrane bound receptors than on soluble receptors. In the case of insulin mutation of site 2 residues (SerA12, LeuA13, GluA17, HisB10, and LeuB17 but not GluB13) produce mutants also with different affinities for the two receptor forms [25]. A mathematical model was recently reported describing the bimolecular reaction between insulin and the IR, and IGF-I and the IGF-1R [33]. It describes first the interaction at site 1 followed by an isomerisation of this low affinity complex to a high affinity complex in which the ligand bridges (“cross-links”) the receptor between sites 1 and 2, with both sites acting in trans from each receptor half. The difference in affinity of mutations at Glu12 for membrane-bound versus soluble receptors could be explained in context of the mathematical model (below). The overall binding affinity is dependent on the rate at which the cross-link is formed (*k*_cr_). An increase in *k*_cr_ results when the receptor is in the soluble form due to removal of the tethering constraint of the cell membrane leading to a higher binding affinity [33]. A decrease in affinity following mutation of the ligand site 2 residues (which are involved in the isomerisation) is more readily detected using membrane-bound receptor where the ability to form the cross-link is reduced due to a lower *k*_cr_ arising from membrane tethering.
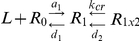
L = ligand, R_0_ = apo receptor, a_1_ = association rate at site 1, d_1_ = dissociation rate at site 1, R^1^ = receptor:ligand complex with binding to site 1 only, *k*_cr_ = cross-linking constant, d_2_ = dissociation of ligand from site 2, R_1×2_ = cross-linked receptor:ligand complex [33].

While the stoichiometry of ligand binding by the IGF-1R and IR is 1∶1 at physiological concentrations there are two potential ligand binding pockets within each receptor's structure. This allows for the characteristic property of accelerated dissociation and negative cooperativity exhibited by both receptors (as described earlier). Here we report the negative cooperativity of IGF-II binding to membrane-anchored IGF-1R. At the concentration range used (up to 1 µM) we observed a sigmoidal dose response curve for negative cooperativity (Figure 3), as is seen for IGF-I on the IGF-1R (membrane bound and solubilised receptor) and IGF-II on solubilised IGF-1R [34], [35]. Interestingly, mutation of Glu12 resulted in a significantly decreased ability to accelerate dissociation, a phenomenon also seen when mutating IGF-I at the equivalent positions to Ala [19], [23]. This is consistent with the notion that mutation of Glu12 decreases the efficiency with which IGF-II bridges receptor sites 1 and 2 and thus implies a key role for this residue in that process.

A major difference in the mechanism of interaction of IGF-II with the IGF-1R and insulin with the IR is the shape of the dose response curve for negativity cooperativity (sigmoidal versus bell-shaped respectively). The ability of the ligand to accelerate dissociation of tracer is dependent on the ability, upon partial dissociation of the first, to form the alternate cross-link (ie binding of another ligand at site 1 and site 2 in the alternate pocket). The reversal of negative cooperativity (which leads to insulin's bell- shaped curve) is thought to be due to two monovalent interactions of insulin at the alternate pocket, one at site 1 and the other at site 2. As the property of negative cooperativity is dependent on interactions at site 2 [19], [36] it is most likely the shape of the curve arises due to subtle differences in the site 2 interaction. Indeed Christoffersen *et al.* [37] demonstrated a bell-shaped curve with IGF-I when using a chimeric IGF-1R with residues 392–579 of the IR (incorporating site 2) inserted into the equivalent region of the IGF-1R. It remains to be determined whether IGF-II is able to induce a bell-shaped dose response curve for negative cooperativity when binding the IR.

The common feature of negative cooperativity and receptor activation is the process of bridging or “cross-linking” the two receptor halves. Consistent with our notion that mutation of Glu12 decreases the efficiency with which IGF-II bridges receptor sites 1 and 2, we see a correlation between a decreased potency in negative cooperativity assays and the ability to activate the receptor by our Glu12 mutants (kinase receptor activation assay). Glu12Ala, Glu12Lys and Glu12Arg IGF-II mutants are also significantly less potent than IGF-II in their abilities to activate phosphorylation of Akt via both the IGF-1R and IR-A. Furthermore, the Glu12Ala, Glu12Lys and Glu12Arg substitutions have a greater than expected decrease in receptor activation compared to their corresponding binding affinities. The disparate receptor binding and activation seen for the IGF-II E12 mutants (and equivalent insulin and IGF-I residues) has not been observed for other IGF-II site 2 residues (Phe19, Leu53, and Glu57) [22]. Therefore, we conclude that the interaction between Glu12 and site 2 residues on both the IGF-1R and IR-A plays an important role in receptor activation by promoting cross-bridging of the two receptor halves and that receptor binding affinity is not necessarily a true indicator of potency in receptor activation. A similar phenomenon was observed when the corresponding residue of insulin HisB10 was substituted with Asp. There were 2 and 5.8 fold increases in IR and IGF-1R binding respectively with a concomitant 2 fold increase in metabolic signalling but an unexpected ∼10 fold increase in mitogenic signalling [31]. It would be interesting in the future to see if the disproportionate effect of the IGF-II Glu12 substitutions on receptor activation leads to different biological outcomes compared to IGF-II.

The current findings suggest that Glu12 of IGF-II and the corresponding residues in both IGF-I and insulin play an important role in receptor activation through their ability to interact with site 2 on the receptors. The cross-bridging of the two receptor halves involving this residue is crucial in the process of receptor activation. Understanding the mechanism of IR and IGF-1R activation is critical for the future design of insulin mimetics and inhibitors of IGF action for the treatment of diseases such as diabetes and cancer.

## Materials and Methods

### Materials and Cell Lines

Long™R^3^IGF-I was purchased from Novozymes GroPep Pty Ltd (Adelaide, South Australia). The DELFIA Europium-labeling kit was purchased from PerkinElmer (Turku, Finland). Europium labeled IGF-II (Eu-IGF-II) was produced according to the manufacturer's instructions and as described by Denley *et al.* [8]. Europium-labeled antiphosphotyrosine antibody PY20 was purchased from Perkin Elmer Life Sciences. P6-IGF-1R cells (BALB/c3T3 cells overexpressing the human IGF-1R) [26] were a kind gift from Professor R. Baserga (Philadelphia, PA). IGF-1R negative (R^−^) cells overexpressing the IR-A (R^−^IR-A) were generated as described [8].

### Construction and Expression of Plasmids Encoding Human IGF-II Glu12 Mutants

The IGF expression vector was developed by King *et al.* [38] and IGF-II cDNA introduced into the vector as previously described [39]. The QuikChange site-directed mutagenesis kit (Stratagene) was used to incorporate codons for Ala, Arg, Asp, Gln, His and or Lys at position 12 of IGF-II (Glu in hIGF-II). The resultant IGF-II mutants were expressed in *E. coli* JM101 (*lac Iq*) or BL21 (DE3) as fusion proteins with the first 11 amino acids of porcine growth hormone ([Met 1] pGH (1–11)) after isopropyl β-D-thiogalactoside induction. Inclusion bodies were isolated as previously described [38].

### Purification of IGF-II Mutants

IGF-II mutants were purified as previously described [40] and shown to have the correct masses by matrix-assisted laser desorption ionization time-of-flight (MALDI-TOF) mass spectrometry (Dr Chris Bagley and Mr Chris Cursaro, Adelaide Proteomics Facility). Purity (>95%) was measured by reverse phase HPLC. All IGF-II mutants maintained the same fold as native IGF-II, as determined by far UV circular dichroism spectral analysis, as previously described [40] (Figure S1). Quantitation of mutants was performed by comparing analytical C4 HPLC profiles with profiles of standard Long™R^3^IGF-I preparations [41].

### Binding Assays

Receptor binding affinities [IC_50_] were measured in two different competition binding assay formats using Eu-IGF-II as the tracer. Whole cell binding assays with P6-IGF-1R and R^−^IR-A cells were performed as described by Alvino *et al.* [22]. Binding to solubilised IGF-1R and IR-A immunocaptured from P6 IGF-1R and R^−^IR-A lysates was as described previously [8], [22].

### Dose-Response Curves for Negative Cooperativity

Dose-response curves for negative cooperativity were performed essentially as described in Gauguin *et al.* (2008) [23] but with some minor modifications. Briefly, serum starved P6-IGF-1R cells (2×10^7^ cells/ml) were incubated with Eu-IGF-II (2×10^6^ counts/tube) in Hepes/BSA (100 mM Hepes, 120 mM NaCl, 5 mM KCl, 1.2 mM MgSO_4_, 8 mM glucose, 0.5% BSA) for 2 h at 16°C. Then 4×10^6^ cells in 20 µl were added to 180 µl ice-cold Hepes/BSA containing increasing concentrations of IGF-II or mutants. After 30 min dissociation at 16°C cells were washed in 400 µl ice-cold tris buffered saline. Cell pellets were resuspended in 100 µl enhancement solution (Perkin Elmer Life Sciences), incubated in the dark for 30 min and then transferred to a white Greiner Lumitrac 600 96-well plate. Time-resolved fluorescence was measured using 340 nm excitation and 612 nm emission filters with a BMG Lab Technologies Polarstar fluorometer (Germany). Assays were performed in triplicate at least three times.

### IGF-1R and IR-A Kinase Receptor Activation Assays

Receptor tyrosine phosphorylation was measured using an adapted kinase receptor activation assay method [42] as previously described [8], [22]. P6-IGF-1R and IR-A cells were stimulated with ligands and phosphorylated receptors in cell lysates were detected with the europium labeled anti-phosphotyrosine antibody PY20 (Perkin Elmer Life Sciences). Assays were performed in triplicate at least three times.

### Immunoblots

P6-IGF-1R and R^−^IR-A cells were treated with 10 nM or 100 nM ligand for 10 min after a 4-hour treatment with serum free DMEM (1%BSA). Cells were lysed in 20 mM HEPES, 150 mM NaCl, 1.5 mM MgCl_2_, 10% (v/v) glycerol, 1% (v/v) Triton-X 100, 1 mM EDTA, (pH 7.5) with freshly added 0.1% (v/v) phosphatase inhibitor cocktail, 2 mM sodium orthovanadate and 10 mM sodium fluoride. Protein concentration was determined with a Bradford protein assay kit (Bio-Rad). Lysates (40 µg) were subjected to reducing SDS-PAGE (7.5% glycine gel). Blots were probed with the polyclonal anti-phospho-Akt S473 (Biosource, Camarillo, CA). Consistency of loading was determined by probing blots with anti-tubulin (Sigma). Blots were stripped in 62.5 mM Tris-HCl (pH 6.8), 2% SDS and 100 mM β-mercaptoethanol for 30 min at 60°C. One-way ANOVA followed by Dunnett's test were used for all statistical analyses. Significance was accepted at P<0.05. Blots were performed at least three times.

## Supporting Information

Figure S1 **Far UV circular dichroism spectra of IGF-II mutants.** The mutants have CD spectra indistinguishable from that of IGF-II. The CD spectra of the mutants Glu12Asp IGF-II, Glu12Gln IGF-II and Glu12Lys IGF-II **A** and of Glu12Ala IGF-II, Glu12His IGF-II and Glu12Arg IGF-II **B** are superimposed on the CD spectrum of IGF-II.
(TIF)

Figure S2
**Activation of the IR-A by Insulin, IGF-I, IGF-II and Glu12Ala IGF-II.** R^−^IR-A cells were serum starved for 4 h and then incubated with increasing concentrations of insulin (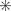), IGF-I (◊), IGF-II (•) and Glu12Ala IGF-II (▴) 10 min. Solubilised IR-A were immunocaptured and phosphorylated tyrosines were detected with Eu-PY20. Receptor phosphorylation is expressed as a percentage of the maximal phosphorylation induced by insulin. The data points are means±S.E. of three assays with each concentration measured in triplicate.(TIF)
