# Supplementary figures and images for: Understanding the Mechanism of Insulin and Insulin-Like Growth Factor (IGF) Receptor Activation by IGF-II
Source: PLoS One. 2011 Nov 28;6(11):e27488. doi: 10.1371/journal.pone.0027488 (PMC3227035; doi:10.1371/journal.pone.0027488)

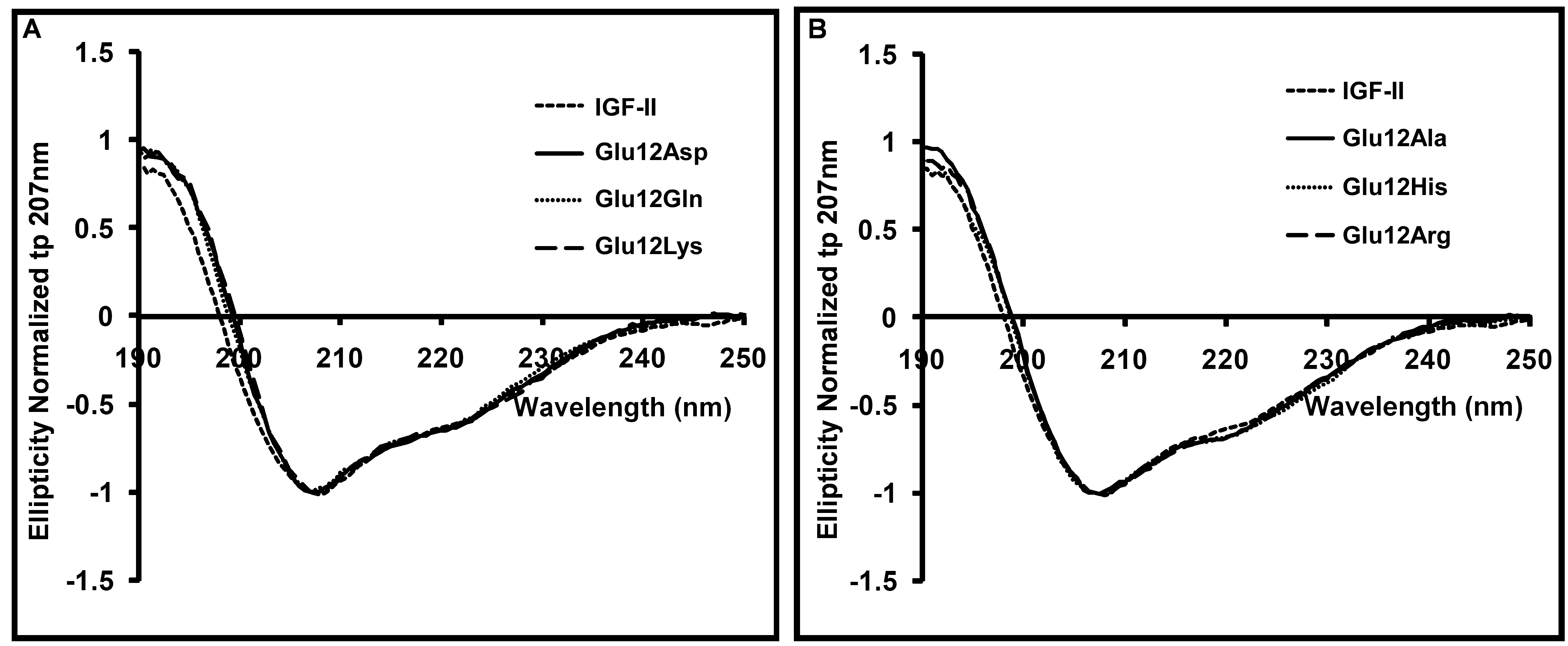

Supplement: Figure S1 — Far UV circular dichroism spectra of IGF-II mutants. The mutants have CD spectra indistinguishable from that of IGF-II. The CD spectra of the mutants Glu12Asp IGF-II, Glu12Gln IGF-II and Glu12Lys IGF-II A and of Glu12Ala IGF-II, Glu12His IGF-II and Glu12Arg IGF-II B are superimposed on the CD spectrum of IGF-II. (TIF) [file pone.0027488.s001.tif]

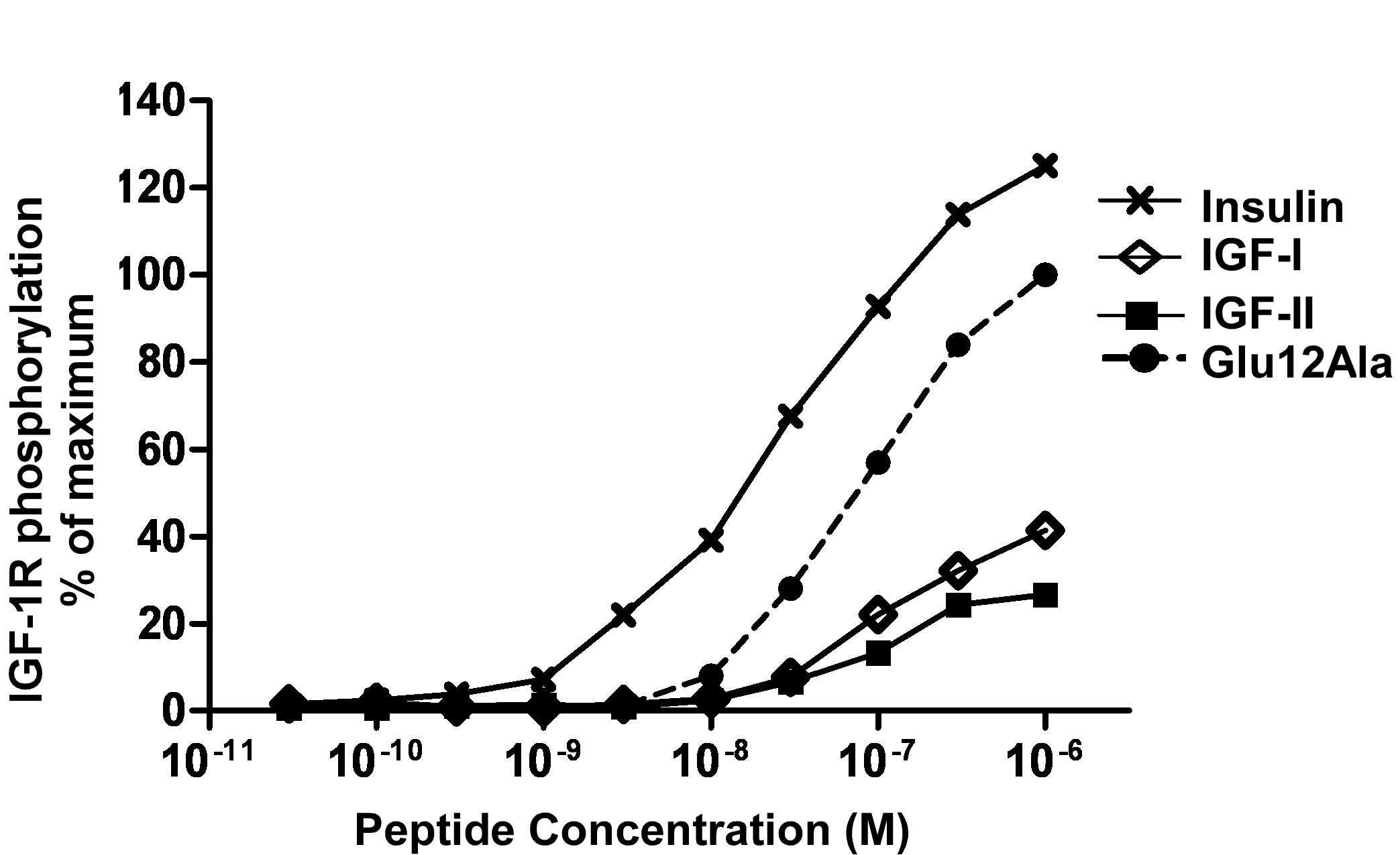

Supplement: Figure S2 — Activation of the IR-A by Insulin, IGF-I, IGF-II and Glu12Ala IGF-II. R−IR-A cells were serum starved for 4 h and then incubated with increasing concentrations of insulin (), IGF-I (◊), IGF-II (•) and Glu12Ala IGF-II (▴) 10 min. Solubilised IR-A were immunocaptured and phosphorylated tyrosines were detected with Eu-PY20. Receptor phosphorylation is expressed as a percentage of the maximal phosphorylation induced by insulin. The data points are means±S.E. of three assays with each concentration measured in triplicate. (TIF) [file pone.0027488.s002.tif]
